# Grief, COVID-19, and the South: Considerations and Recommendations

**DOI:** 10.1177/00302228211029500

**Published:** 2021-07-01

**Authors:** Danielle L. McDuffie, Kathryn Kouchi, Hillary Dorman, Elizabeth Bownes, Shelley E. Condon, Martha R. Crowther

**Affiliations:** 1 Department of Psychology, 8063 The University of Alabama, The University of Alabama, Tuscaloosa, Alabama, United States; 2 College of Community Health Sciences, 8063 The University of Alabama, The University of Alabama, Tuscaloosa, Alabama, United States

**Keywords:** COVID-19, bereavement, grief, rural, South

## Abstract

**Purpose:**

COVID-19 has devastated the United States (U.S.). One of the more notably impacted areas is the South. Compared to the rest of the U.S., the South is characterized by increased rurality, lowered access to healthcare, older populations, and higher religiosity, all of which might predispose its residents to more detrimental effects of COVID-19, including COVID-related fatalities. As such, this paper provides important considerations for individuals engaging in work with Southern, rural Americans dealing with COVID-related grief and loss.

**Methods:**

A review of the literature addressing the impact of Southern legislature, rurality, cross-country factors, and faith on COVID-related grief among Southerners was conducted, with applicable considerations expressed.

**Conclusions:**

Care should be taken by providers working with rural, Southern residents to attend to tangible and intangible losses experienced as a result of COVID-19. These considerations can help inform work with rural Southerners dealing with grief during the pandemic.

Coronavirus, also distinguished by the moniker COVID-19, has ravaged the United States over the last year. While the initial U.S. epicenter of the virus was in the Northeastern United States, a growing area of concern for the spread of the disease emerged in the South over the late Summer and early Fall months. Reports from the end of June throughout the months of July and August showed growing trends of positive COVID-19 cases in the South at troubling rates (Bendix & Woodward, 2020; Bredemeier, 2020; Laughland, 2020; Stone, 2020). States such as Alabama and Texas were showing staggering increases in weekly rates of new cases during mid-to-late June (97% rise in weekly cases and 130% rise in biweekly cases, respectively) (Bendix & Woodward, 2020; Stone, 2020). At present, rises in COVID-19 infections and fatalities have been identified all over the country. While the South is no longer showing infection rates and deaths at a disproportionately higher rate than other parts of the country, Southern states such as Texas and Florida continue to be among states with the highest surges in COVID-19 related deaths, with each state seeing at least 500 deaths in a single week as of the first week of January (*New York Times*, 2021). Additionally, Southern states such as Georgia and Mississippi are high on the list of states struggling with the roll-out of the country’s COVID-19 vaccine (*New York Times*, 2021).

The rise and sustained high number of COVID-19 cases and fatalities in the South, particularly during the late Summer and early Fall, have been attributed to multiple factors. One is the slower timeline by which many Southern legislators enforced COVID-19 restrictions within the early Fall months. Additional factors include the premature laxing of restrictions during the late Summer and early Fall, non-enforcement of mandates on mask wearing, and a lack of social distancing (Stone, 2020). Ensuing death as a result of COVID-19 is something that warrants particular consideration in the South. The South, compared to other parts of the country, has multiple factors that might predispose its residents to a higher prevalence of COVID-related loss. This consideration also makes Southern residents more susceptible to grief and negative bereavement-related outcomes.

The South is characterized by a few factors that distinguish its population from other areas in the country and highlight potential predispositions to COVID-related distress. Initially, it has been found that COVID-19 disproportionately affects older adults. Relative to this, the South has a slightly older population than other areas of the U.S. Of the states with the highest populations of adults aged 65 and older in 2018,2 of the top 3 states were located in the South (Florida and Texas; U.S. Census Bureau, 2019). Moreover, COVID-19 has been found to have a greater impact in populations with health disparities. Chronic diseases, immunocompromising illnesses, and cancer mortality rates are all higher in the South (Kaiser Family Foundation, 2018a; National Academies of Sciences Engineering Medicine, 2013). Chronic diseases such as hypertension may be linked to COVID-related hospitalizations, which is significant considering Southern states comprise the top ten states diagnosed with hypertension in terms of population percentages (KFF, 2018a). Structural factors such as poor access to healthcare in some of the more “deep South” states have led to disproportionately high rates of infection and death, particularly among minority populations (Laughland, 2020). Research attributes several factors to these health disparities, including significant differences in poverty levels and policies, as well as systemic racism (Kaiser Family Foundation, 2018b). For example, Southern states comprise nine of the fourteen states who refuse to expand Medicaid to impoverished residents under the Affordable Care Act (KFF, 2018b). Another factor that is unique to the South is increased religiosity. Residents of the South tend to be more religious than in other parts of the country, with the Southwest and Southeast containing 9 of the 11 most religious states in the nation (Norman, 2018). Many religious individuals engage in the practice of congregating to express their faith. This form of congregation also extends to religious celebrations commemorating the loss of a loved one (BBC, 2009). The tendency to congregate is highly incompatible with the safety recommendations for the prevention of the spread of COVID-19. The incongruence between the desire to congregate among religious individuals and mandates of distancing could also cause exacerbated rates of distress among Southern populations. Summarily, an older population, poorer healthcare outcomes, and higher religious involvement make the South a region extremely vulnerable to higher COVID-19 infection rates and COVID-related fatalities. It is with this information that we seek to address considerations for the impact of COVID-19 on the grieving process, particularly amongst residents of the South.

In distinguishing the terms relevant to the loss of life, it is important to differentiate between grief, bereavement, and mourning. Grief has been defined as disruptions in functioning during the early period following the loss of a loved one (Bonanno & Kaltman, 2001). Bereavement is a normative stressor characterized by the death of a loved one (Bonanno & Kaltman, 2001). Mourning, in its distinction from the prior terms, is the temporary social role assigned to grief whereas grief becomes publicly recognized and expressed through action (Charmaz & Milligan, 2006). Succinctly, grief is the internalized reaction to bereavement, and mourning is the outwardly expressive form of grieving. The purpose of this review was to address the impacts of COVID-19 in the South and the resulting implications on grief and bereavement outcomes within this unique population. For the purposes of the present considerations, we will address factors related to the processes of grief and mourning on individuals residing in the South who are likely to be affected by COVID-19.

## Methods

The present manuscript is a review of the current literature on COVID-19 and bereavement in the South. We modeled this review loosely in accordance with the integrative review guidelines proposed by Whittemore and Knafl (2005), positing the necessity of 1) identifying a problem, 2) searching the literature, 3) evaluating the data, 4) analyzing the data, and 5) presenting the results. In our identification of a problem, we drew on our personal experiences. All of the authors are clinicians (either trainee-level or licensed) working within a Southern state. Within our clinical work, a multitude of our clients were struggling with implications not only of COVID-19, but specifically of handling multiple losses at a professional and/or personal level.

In conducting a more thorough search of the existing literature surrounding COVID-19 and its effects, the keyword “COVID-19” was used. Literature searches were conducted on research databases and Google Scholar to find articles describing the state of COVID-19 in the South. After finding literature addressing impactful factors that might exacerbate the role of COVID-19 in the South, additional literature searches were conducted through databases to find information concerning trends of rurality, religious faith, and cross-cultural factors impacted by COVID-19. Articles that were excluded from the initial keyword search did not address COVID-19 specific to the South.

## Data Evaluation

The final sample of papers used within this review included empirical and opinion-based articles. This was due to the novel nature of COVID-19 and the constantly shifting literature. Considerations for the present manuscript were formed through amalgamations of COVID-19 trends in the South with factors unique to the South that were impacting rates of infection and mortality from COVID-19. These factors were garnered from the investigative and current empirical literature from leading scientific organizations (i.e. the Centers for Disease Control and Prevention [CDC]) addressing the spread of COVID-19 in the South. Based on these considerations and the observed problem, recommendations for healthcare workers from physical, mental, and behavioral health disciplines were deduced. It should be noted that data used in preparation of this article is provisional and incomplete, which may cause conclusions to change over time.

## Considerations

### Southern Response to COVID-19

Although COVID-19 has spread ubiquitously throughout the United States, the most recent trends show a sustained amount of high case numbers in the Great Plains and the South relative to other parts of the country (CDC, 2021; Treisman, 2020). In the early stages of the pandemic, populous states and big cities were more likely to show higher infection rates. However, during the late Summer and early Fall months, the *New York Times* (2020) reported that available data identified the Southwest as having the most cases per capita. While there was speculation about travelers from the North being the reason for increasing COVID-19 rates in the South, an analysis by the Harvard Global Health Institute (2020) attributed the large outbreaks to states’ decisions to prematurely relax COVID-19 regulations in May and later allow students to return to school (or return without adequate procedures in place) in August.

This proved especially concerning for infection rates at Southern colleges, which comprised six of the top seven states with the most reported campus cases of COVID-19 during the late Summer and early Fall months (Texas, Georgia, Alabama, North Carolina, South Carolina; *New York Times*, 2020). Infection rates and case-fatality among younger and middle-aged adults were found as being significantly higher among Southern states (Deep South and Mid-South) as compared to other regions, where people aged 70 and older are most susceptible (Newkirk, 2020). For example, the 40 to 59 age group accounted for 22% of COVID-19 deaths in Louisiana and 6% of COVID-19 deaths in Washington by the early Fall (Newkirk, 2020). Additionally, with wide re-openings followed by subsequent shutdowns and holiday breaks, the likelihood for Southern college students to travel to other parts of the country to return home had the capacity to lead to high rates of infection and mortality amongst middle to older aged family members and those with compromised immune systems.

### Impacts of Rurality

A notable percentage of U.S. Southern geography is rural. One in five Americans live in rural areas. These rural communities represent a considerably vulnerable population for COVID-19 morbidity and mortality risk (Peters, 2020). COVID-19 has been associated with age (80% of COVID-19 deaths were individuals aged 65 years or older), underlying health conditions (90% of COVID-19 hospitalized patients), and limited access to necessary health care services (Leroy et al., 2020). Compared to urban counterparts, rural communities represent a demographic that is predominantly older, with higher rates of chronic physical (eg, diabetes, heart disease) and mental (eg, substance use, suicide rates) health conditions, higher rates of disability risk, and limited access to healthcare services (CDC, 2020a). Additionally, while it is known that COVID-19 poses a larger risk among those with serious health conditions, health disparities (eg, obesity, heart disease, and lung disease) and poorer health outcomes are far more common within the South compared to the rest of the U.S. (Koma & Neuman, 2020). Furthermore, over the past decade, rural health systems have faced under-resourcing, resulting in more than 100 hospital closures. Sixty percent of these closures have occurred in the South (Stabenow & Manchin, 2020). In the South, minority groups have less access to and spend less on public health annually (Koma & Neuman, 2020). The circumstances by which rural residents were lacking in healthcare provisions were already dire; however, these strains have been further exacerbated by the pandemic.

Death during the COVID-19 pandemic presents unique challenges that can disrupt grieving processes. A noted protective factor in the grieving process is social support. COVID-19 deaths, however, have been described as lonely and dehumanizing for patients and families (Leroy et al., 2020). Within rural communities, this social separation is magnified by limited access to high-speed internet (only 26% of rural Americans have access; Lyne et al., 2020; Stabenow & Manchin, 2020). It is known that during these times, many bereaved individuals have looked to internet channels to engage virtually with their ill loved ones and/or to engage in services for deceased loved ones. With large parts of the South being rural, it can stand that many of the people residing in the South are suffering both from a higher susceptibility to COVID-related loss and less accessibility to practices that ordinarily aided with their grief. The visible and invisible barriers for rural residents can significantly interfere with their ability to grieve and effectively cope with their loss. Focusing on grief in rural regions is essential, as grief-related coping encompasses cultural values, beliefs, and practices specific to the individual and their contextual environment. This is imperative for those providing care to bereaved Southern residents to be attentive to, particularly during the ramifications from COVID-19.

### Cross-Country Factors

Grieving from a distance is fraught with challenges, but amidst a pandemic, experiencing the loss of a loved one in another state or part of the country is additionally difficult. One of the first lines of defense against the spread of COVID-19 internationally was to restrict and limit travel (Lau et al., 2020). As cases rose in the United States, the Center for Disease Control (CDC) implemented a number of public health measures including maintaining a social distance of 6 feet from those not in one’s household, canceling large gatherings of people and events, and implementing an initial two-week lockdown where individuals were not to leave their home for non-essential tasks (CDC, 2020b). Travel to other areas of the country was strongly discouraged, with each state having varying levels of “openness” to visitors. While some states were open to out-of-state guests, others required a two-week quarantine upon arrival (CDC, 2020b). Compounding this was the fact that most states strongly discouraged residents from the South from travel, and this is a trend that has continued through present. This culmination of factors provides a notable challenge to Southern residents with family members in other parts of the country or world who have recently died.

The ever-changing landscape of the pandemic poses the question of the importance of participation in bereavement services for the wellbeing of the bereaved individual. Southern residents who experience the loss of a loved one living elsewhere must balance the desire to be near loved ones during this time with fears of spreading or contracting COVID-19 or facing quarantines. A rapid review of the literature by Burrell and Selman (2020) found no conclusive support for improved mental health outcomes relative to the participation in funeral practices. However, qualitative research supported the benefit of active engagement in meaningful funeral and after-death practices. Mourners during the pandemic are required to be more intentional about their participation in funeral rituals. Particularly for some of those in the South, bereaved family members may elect not to travel and to instead utilize technology, such as FaceTime or other video conferencing services to engage in funeral practices from a distance. However, as noted earlier, this is not a luxury available to all Southern residents, particularly those residing in more rural areas (Lyne et al., 2020; Stabenow & Manchin, 2020). The pandemic has impacted every aspect of life, and for family members grieving from afar, we must be mindful of their needs and desires to process their loss in a meaningful way.

### Religion and Spirituality

Religion and spirituality are an integral part of life in the South. Mississippi and Alabama are consistently ranked as the two most religious states (Newport, 2017), reflecting the high number of churches per capita and percentage of residents endorsing religiosity and church attendance. Religion and spirituality provide a daily and weekly routine, a place of worship, social connection and community (Lim & Putnam, 2010), a value system, sense of purpose (Koenig, 2015), comfort with uncertainty (Hogg et al., 2010), belief in the afterlife, and rituals for death and dying. Additionally, religiosity positively impacts health and well-being (Cohen & Johnson, 2017; Cohen & Koenig, 2003; Koenig, 2012).

However, there are important caveats to consider when addressing COVID-related grief among Southern populations. While religious faith (e.g., belief in a loving, protective God, the afterlife, etc.) can cultivate strength and lessen death anxiety (Flannelly, 2017; Harding, Flannelly, Weaver, & Costa, 2005), it may also contribute to the spread of COVID-19 (Dein, Loewenthal, Lewis & Pargament, 2020). Recent research suggests that Christian nationalism and religiosity may lessen adherence to public health protocols, like wearing a mask and avoiding large social gatherings (Perry, Whitehead, & Grubbs, 2020). With the association between religiosity and the South, it is important to consider the role of one’s religious background when interacting with and providing care to a Southern resident dealing with loss subsequent to COVID-19. Recognizing the multifaceted role that religion and spirituality play in adapting to life and loss during COVID-19 is important for those providing services to individuals residing in the South during these times.

## Recommendations

The following recommendations address the nuances of COVID-19 grief within Southern, rural communities to mitigate distress, recognize vulnerability, and promote resiliency during these uncertain times. These recommendations are directed towards physical, mental, and behavioral healthcare workers.

- Help patients to engage in sensitive, yet direct discussions regarding end-of-life planning. (These discussions, while sometimes uncomfortable, can help to mitigate future distress at the end of life, both for the bereaved individuals and those concerned about the effects of their loss on their loved ones).
- Validate the emotions of patients, both during the grieving and end-of-life decision making processes.
- Aid patients in accessing social support networks in a manner sensitive to COVID-19 restrictions. (This could require consultation with Social Work staff members or outside resources).
- Suggest to patients the necessity of adapting mourning practices to accommodate COVID-19 requirements. (This could include engaging in indirect socialization and mourning such as writing letters, making art, or participating in virtual ceremonies/support groups).

## Conclusion

COVID-19 has impacted rural and remote areas in significant ways and poses unique challenges during these times (Kotani & Manabe, 2020). Rural communities tend to prioritize relationships that can provide valuable support during grieving and bereavement processes. However, in a time of social distancing and self-isolation, this social support may look different or be lacking. Grief and mourning practices look different during COVID-19, particularly for our older, more religious, and more rural Southern neighbors. Understanding the differences and unique challenges posed by the pandemic for bereaved individuals is important for all groups, but especially for the rural areas and areas of the South with reduced access to mental health support or resources. In a time composed of a great deal of loss and despair, it is important that we are mindful of the unique needs of all individuals and continue to develop ways to provide effective aid and maximally efficacious care.
